# American Dental Society of Europe

**Published:** 1873-10

**Authors:** C. M. Wright

**Affiliations:** Basle, Switzerland


### AMERICAN DENTAL SOCIETY OF EUROPE.

On the 4th of July, 1873, a few enthusiastic American dentists of Switzerland met at Lucerne, and made the trip of the Rigi; and while on the little steamboat, on the lovely lake, surrounded by the frowning Pilatus, the stately Rigi and the snow-capped Alps, discussed the plan of establishing an "American Dental Society of Europe."

The object of the meeting was to celebrate the Fourth, and "talk" about founding a society. The "talk" was simply earnest with confident determination to form the first "American Dental Society of Europe," and when the top of the Rigi was reached, all that was necessary was to elect officers for the year, and resolve to devote our energies to the object.

The above name was given to the society, and the following officers elected:

*President.*—Dr. C. T. Terry, of Zurich.

*Vice-President.*—Dr. J. G. Van Marter, of Basle.

*Recording Secretary.*—Dr. C. M. Wright, of Basle.

*Corresponding Secretary.*—Dr. N. W. Williams, of Geneva.

*Treasurer.*—Dr. G. W. Field, of Geneva.

*Executive Com.*—Drs. Van Marter, Field and Williams.

After the election of officers, the session was passed in informal discussions of the practical topics of our profession, and though no report can be offered, the general tone was advocacy of the advanced methods of operating, discussion about the character of cases presented here in Europe, etc. We hope by next year to offer valuable results of observations made in this Old World.

Six thousand feet above the sea, with a magnificent panorama of lake and mountain scenery, and in view of the spot where Swiss liberty was born, the first "American Dental Society of Europe" was born. May she ever maintain her *elevated* position. May she hold her members as high, and may her lofty example raise the standard on every mountain-top in this country.

We ask for the sympathy and recognition of our much-loved American dental associations. As three or four of the officers have been intimate in the old "Mississippi Valley," the "Mad River Valley" and the "Ohio State" societies, we ask for their sympathetic support. The time of the next meeting, and the topics, will be reported in due time.

C. M. Wright, D. D. S., *Recording Secretary.*

Basle, Switzerland, July 5, 1873.

